# Benign paroxysmal positional vertigo: A case report in which four wrongs made a right

**DOI:** 10.1002/ccr3.9529

**Published:** 2024-11-05

**Authors:** Lisa Young, Shervin Badihian, David S. Zee, David E. Newman‐Toker, Kevin A. Kerber, Anita Bhandari, Rajneesh Bhandari

**Affiliations:** ^1^ Department of Neurology Johns Hopkins University School of Medicine Baltimore Maryland USA; ^2^ Neurological Institute Cleveland Clinic Cleveland Ohio USA; ^3^ Department of Otolaryngology‐Head and Neck Surgery and Ophthalmology Johns Hopkins University School of Medicine Baltimore Maryland USA; ^4^ Department of Neurology Ohio State University Columbus Ohio USA; ^5^ Vertigo and Ear Clinic Jaipur India; ^6^ NeuroEquilibrium Diagnostic Systems Pvt Ltd. Jaipur India

**Keywords:** BPPV, Epley maneuver, lateral canal, positional nystagmus, positional vertigo

## Abstract

Repositioning maneuvers for benign paroxysmal positional vertigo (BPPV) designed to induce otoconial movement in one canal can trigger and sometimes unwittingly treat BPPV in other canals. Patients with BPPV are best managed by precisely diagnosing the canal variant and using correctly performed, standardized testing and treatment maneuvers.

## 1 INTRODUCTION

Benign paroxysmal positional vertigo (BPPV) is a common cause of episodic vertigo in which otoconia (calcium carbonate crystals) become detached from their usual location on the macula of otolith organs.^1^ They can then find their way into the semicircular canals, become free‐floating, or attached to the cupula, and create abnormal sensations of motion and nystagmus when the position of the head changes with respect to gravity.

BPPV of the lateral canal (lcBPPV) is less commonly diagnosed than BPPV of the posterior canal BPPV (pcBPPV), possibly because of confusion in distinguishing canal variants in clinical practice.^2^, ^3^, ^4^, ^5^, ^6^ The supine roll test (SRT) and the Dix‐Hallpike test (DHT) are primarily used to diagnose lcBPPV and pcBPPV, respectively.^7^, ^8^

BPPV is usually treated with particle repositioning maneuvers that move the otoconia out of the semicircular canal: the Epley or Semont maneuvers for pcBPPV and, for example, the Gufoni, Zuma, or Lempert (barbecue roll) maneuvers for lcBPPV.^7^, ^9^, ^10^, ^11^ Although the Epley maneuver has been anecdotally reported to resolve lcBPPV, limited research has been performed to establish a mechanism by which this outcome can occur. Understanding this mechanism is important because it may optimize clinicians' ability to treat patients with lcBPPV.

## 2 CASE HISTORY/EXAMINATION

A 19‐year‐old woman with no past medical and surgical history came to the emergency department around noon because of a single episode of acute vertigo with unsteadiness and light‐headedness, which was triggered by changing her head position. Her spell began on awakening around 3:00 a.m. that same day. Her symptoms partially subsided when lying down in bed and not moving. She denied headaches, photophobia, vomiting, diarrhea, recent viral illness, medication use, head injury or trauma, hearing loss, or migraines. She was otherwise healthy. She had a similar prior episode of vertigo that resolved on its own after a nap; however, exact details were not documented at the time of the emergency department visit.

### 2.1 The sequence of events in the emergency department

While in the emergency department, she was enrolled in a diagnostic clinical trial (clinicaltrial.gov identifier: NCT02483429) approved by the institutional review board of the participating institution to investigate the use of video‐oculography (VOG) testing in the initial evaluation of acutely dizzy patients in the emergency department. This was a single‐blind (to outcome assessors only) clinical trial, and the blinding was broken after the trial was completed. As part of this clinical trial, our research staff obtained a VOG study *before* the emergency department physicians evaluated the patient. Participants were then randomized into two arms. In the standard‐of‐care arm, patient records and VOG findings are not reviewed in real‐time by anyone from the study, and their emergency department physician team was unaware of the VOG results. In the intervention arm, a neurologist from the study reviewed a patient's VOG and symptom profile and suggested a diagnosis. This diagnosis and management recommendations were shared with the emergency department physician; however, it was the physician's ultimate decision whether they wanted to follow them. In other words, it acted like a consult service. The study protocol did not involve repeat VOG testing after treatment in the emergency department.

Our patient was randomized into the intervention arm, so the VOG findings and consultant recommendations were shared with the emergency department physician team. At that time, the on‐call clinical trial neurology fellow felt they could not rule out a central etiology based on VOG traces, so they recommended further neuroimaging (brain MRI), a neurology consult, and possibly admission to the hospital. The emergency department physicians, however, felt the presentation was more consistent with a peripheral etiology.

Their emergency department physicians performed an independent physical examination and vestibular assessment to rule out other causes of vertigo. They noted horizontal nystagmus with leftward gaze when the patient was sitting up. Hearing was evaluated with self‐assessment and with the calibrated finger rub auditory screening test (CALFRAST). No subjective hearing loss was noted, and the best finger rub was heard at 70 cm in both ears. Neither a neurology consultation nor neuroimaging were obtained, and the emergency department physicians made the final diagnosis.

## 3 DIFFERENTIAL DIAGNOSIS

The differential diagnosis for vertigo includes peripheral (BPPV, Meniere disease, vestibular neuritis, labyrinthitis, aminoglycoside toxicity, perilymphatic fistula, etc.) and central etiologies (stroke, posterior fossa tumors, vestibular migraines, demyelination, etc.).^7^ The only aforementioned condition she met criteria for was BPPV.

## 4 INVESTIGATIONS

During the VOG study, visual fixation was suppressed using ICS Impulse goggles (Otometrics, Natus Medical, Inc., Pleasanton, California). Otosuite Vestibular software (version 4.4, build 1459) was used to analyze the VOG recordings. VOG results are described in Table 1. Video S1 shows the VOG recording of the right supine roll, and Video S2 shows the VOG recording of the left supine roll.

**TABLE 1:** Video‐oculography testing results.

Position or maneuver	Results
Video head impulse testing for lateral canals	Gains within normal limits without corrective saccades (head left, 0.83; head right, 0.97)
Sitting upright at center gaze	Slow right‐beating spontaneous nystagmus of 1–2°/s
Supine	Right‐beating spontaneous nystagmus of 3°/s
Chin‐down (head bowed position)	Right‐beating sustained horizontal nystagmus (max SPV of 7°/s)
Right DHT	No nystagmus
Left DHT	Sustained right‐beating horizontal nystagmus (max SPV of 12°/s
Right supine roll	Crescendo‐decrescendo right‐beating nystagmus, lasting ~40 s (max SPV of 12°/s)
Left supine roll	Briefer decrescendo left‐beating nystagmus, lasting ~15 s (max SPV of 13°/s)

Abbreviations: DHT, Dix‐Hallpike Test; SPV, slow‐phase velocity.

The emergency department diagnosis was obtained after the emergency department physician performed a DHT. They diagnosed a *left‐sided* BPPV, presumably of the *posterior canal*, though the details of any elicited nystagmus were not reported. After the emergency department visit, the research team's expert panel reviewed the VOG findings per the research protocol. The panel determined that the overall picture was most consistent with the *geotropic variant of lc*BPPV (i.e., a positional nystagmus in which quick phases beat toward the ground in both ear down positions). Based on the intense right‐beating nystagmus induced in the head bowed down position, it was likely a *right‐sided* lcBPPV.^12^

## 5 TREATMENT, OUTCOME, AND FOLLOW‐UP

The emergency department physicians (who were not privy to the expert panel's final diagnosis) treated her with a *left‐sided Epley* maneuver, which was repeated twice. During the first Epley maneuver, the patient developed vertigo when lying with her head turned to the left. The vertigo stopped after she reached the right ear down position, and her head had been pointed to the floor for 2 min. There was no vertigo with the second Epley maneuver. The emergency department physician documented the patient's symptoms and nystagmus as resolved. She was discharged directly from the emergency department, feeling well. Unfortunately, the study personnel were unable to reach the patient via phone or mail for the in‐person follow‐ups at 1 week, 1 month, and 6 months. Our research team could not collect further follow‐up data due to the expiration of the clinical trial window to contact patients.

We asked why the seemingly wrong treatment worked, so we performed simulations to test different possible treatment maneuvers using a 3D model of the inner ear. This generic model was based on reconstructed images from high‐resolution computed tomography of the temporal bone Digital Imaging and Communication in Medicine (DICOM) files. Bhandari and colleagues have previously described this methodology.^13^ The orientations and angles between the canals were as previously reported.^14^ The simulations allowed debris placement in any of the canals and at variable positions within the canals. Given the uncertainty of whether the emergency department physician correctly performed the Epley maneuver on this patient, we simulated both possibilities: a correctly performed Epley maneuver and an extended Epley maneuver.

Simulation 1 (Video S3) shows the effect of a correctly performed left‐ear Epley maneuver on a right‐ear lcBPPV. When the subject is brought to the head‐hanging position in the second step of the maneuver, the debris moves (under the influence of gravity) through but not out of the lateral canal away from the ampulla. This could generate a left‐beating horizontal nystagmus. When the subject's head is turned 90° to the right side, the debris moves back through the lateral canal toward the ampulla and could generate a right‐beating horizontal nystagmus. Thus, the correct Epley maneuver with a lcBPPV of the opposite side causes free‐floating otoconia to move within but not out of the offending lateral canal. The patient's lcBPPV symptoms would likely not be resolved. This simulation also applies to the second step of a correctly performed DHT.

Simulation 2 (Video S4) shows the effect of a left Epley maneuver when the head is turned by 15° more than the usual 45° in the second step of the maneuver in a right lcBPPV. When the subject is brought to the head‐hanging position, the contralateral lateral canal becomes nearly vertical, allowing the debris to move not only within but also out of the right lateral canal under the effect of gravity. This would successfully treat a lcBPPV on the contralateral side before the patient is rolled over. This simulation also applies to the second step of an extended DHT.

## 6 DISCUSSION

This case report highlights the successful treatment of right‐sided BPPV of the lateral canal (lcBPPV) in a young woman despite diagnosing the wrong side and the wrong canal, applying the wrong treatment maneuver, and wrongly performing the treatment maneuver.

### 6.1 Diagnosis

LcBPPV is often underdiagnosed in favor of pcBPPV.^2^, ^4^, ^5^, ^6^ Several theories have been proposed in the literature to explain this. Firstly, otoconial debris are less likely to become trapped in the lateral canal than the posterior canal due to the spatial orientation of the semicircular canals. The lateral canal has an upward slope and a cupular barrier at the upper end, which is more conducive to allowing debris to flow through the non‐ampullary tract of the canal back into the utricle.^15^, ^16^, ^17^ Additionally, spontaneous resolution is more common in lcBPPV compared to other canal variants.^18^ Another contributing factor may be that many emergency department and primary care providers do not attempt to distinguish canal variants.^3^

LcBPPV can be distinguished from pcBPPV based on several clinical characteristics, most notably the direction of its nystagmus. The former produces horizontal nystagmus and the latter a vertical/torsional nystagmus, in accordance with Ewald's 1st law (“the plane of nystagmus parallels the anatomic plane of the semicircular canal that generated it”).^8^ The supine roll test (SRT) is the preferred test to diagnose lcBPPV, and the Dix‐Hallpike Test (DHT) is the preferred test to diagnose pcBPPV.^7^ However, the DHT can cause vertigo and horizontal nystagmus in patients with lcBPPV, which may be confused with pcBPPV.^19^, ^20^ Moreover, alterations of the angle in which the head is positioned relative to gravity may reduce the diagnostic accuracy of the DHT.^21^ This is consistent with the analysis of the response of our patient, which showed that the DHT could cause debris to move within the contralateral lateral canal, presumably producing symptoms and potentially leading to a misdiagnosis of pcBPPV instead of lcBPPV. Note there is often a “pseudo‐spontaneous nystagmus” in lcBPPV, which could have accounted for the weak spontaneous nystagmus on center gaze and in the supine position shown by our patient. However, because of its low intensity, it could also be within normal limits.^22^, ^23^

Current guidelines recommend performing the SRT to assess for lcBPPV if the patient's history is compatible with BPPV and DHT shows horizontal or no nystagmus.^6^, ^7^, ^8^, ^11^ The potential for misdiagnosis, especially if the DHT is not correctly performed, raises the question of whether the order of these diagnostic tests should be changed. One possibility is that providers could first perform the SRT in patients with BPPV because the SRT would trigger little to no movement of particles within the posterior canal.^24^ Even if there was movement in the posterior canal, it would produce vertical/torsional nystagmus pointing to the posterior canal being affected. Bhandari et al. (2022) have further shown with simulations that the SRT itself may move debris out of the canal.^24^ If the SRT is negative, providers could perform the DHT to assess for pcBPPV. Alternatively, providers could be trained to start with the DHT but, more specifically, accurately recognize nystagmus patterns of pcBPPV or lcBPPV since the DHT has been reported to trigger nearly all pcBPPV and at least half of lcBPPV.^20^ However, modifications to diagnostic protocols must be balanced with the need to be efficient and cost‐effective, especially in emergency department or primary care settings. Future clinical trials may be helpful in determining whether these modified diagnostic approaches can improve the accuracy of BPPV canal subtype classification in these settings.

### 6.2 Treatment

The “curative” effect of the extended Epley maneuver in this case was surprising because, if correctly performed, it should be specific for resolving pcBPPV.^7^ There have been anecdotal reports that lcBPPV may resolve with the Epley maneuver, but it is unclear how the Epley maneuver was performed in those cases.

We speculate this patient's successful outcome occurred because it was anatomically plausible. The greater head rotation to the left (the healthy side) in the extended Epley maneuver could have allowed the debris to move out of the right lateral canal under the effect of gravity. Alternatively, canal switching of lcBPPV into pcBPPV could have occurred during repositioning maneuvers, which would explain her successful treatment with the Epley maneuver.^25^

This extended Epley maneuver is similar to a proposed treatment maneuver for lcBPPV that involves starting in the supine position, turning the head toward the healthy side for 45°, then another 45° toward the healthy side, and then sitting up.^26^ Another study proposed turning the head toward the healthy side at a downward 120° angle from supine.^27^ Although this study hints that modified or universal repositioning maneuvers may be practical to treat multiple canal variants, further studies are needed to evaluate and compare their efficacy.^28^ The optimal repositioning maneuvers for lcBPPV and pcBPPV are well‐documented and should be encouraged in clinical practice.^7^, ^8^

### 6.3 Clinical implications

The importance of the precise diagnosis of the side affected and the canal subtype for successful treatment of BPPV is well‐established,^7^, ^11^, ^17^ and the “lucky” results in our patient should not be interpreted to suggest that clinicians need not strictly follow standardized protocols when performing the DHT or Epley maneuver. Indeed, our results suggest the *opposite*—that placing the head in the correct position for a canal‐plane‐specific BPPV is critical to interpreting nystagmus and ensuring accurate clinical diagnosis. Failure to do so may lead to treatment with an inappropriate repositioning maneuver, leading to unpredictable and unsuccessful outcomes. Given the high recurrence rate and dizziness handicap associated with lcBPPV, education of emergency department and primary care providers is critical to ensure accurate diagnosis and treatment of patients presenting with dizziness.^29^

### 6.4 Limitations

Our study had several limitations. The first was the lack of follow‐up data on our patient's long‐term response given the expiration of the clinical trial window for contacting patients. Despite the emergency department physicians describing the resolution of symptoms and nystagmus upon discharge, the lack of follow‐up means it remains uncertain whether the patient had a complete or partial treatment response to the incorrect repositioning maneuver. Additional limitations include the lack of precise knowledge of how the clinicians performed testing and treatment maneuvers in the emergency department and the lack of precise knowledge of the nystagmus patterns observed by the treating clinician. Furthermore, since there was only a small difference in the intensity of the horizontal nystagmus elicited in the two ear‐down positions during the SRT, the localization of the offending labyrinth by the research team was primarily based on the response to the bow test. The lack of significant asymmetry between the two sides during the SRT could be related to the relatively low peak slow phase velocity (SPV) of nystagmus induced in the SRT, which could have been due to the relatively low velocity of the movements of the head to the ear‐down position.^30^, ^31^ In this case, the head movements were not fast enough for Ewald's 2nd law—excitation produces a stronger response than inhibition—to take effect. The asymmetry between right and left ear‐down positions may be better brought out by moving the head quickly to the ear‐down positions. Finally, our simulations assume an average anatomical orientation of the canals, membranous labyrinth, and cupula.^14^ The variability among individual subjects of the orientation of the canals in the head, the membranous labyrinth within them, and the cupula; the presumed differences among subjects in the location and constitution of the debris within canals; and the variability in the speed at which the head is brought to a new position in diagnostic and treatment maneuvers, can all contribute to the uncertainty of the classification of the canal subtype of BPPV and the variable response of different treatment maneuvers.^1^, ^32^, ^33^, ^34^

## 7 CONCLUSION

Repositioning maneuvers designed to induce the movement of displaced otoconia in one specific canal can trigger and treat BPPV in other canals. We recommend that clinicians: (a) confirm that the head is placed in the correct position for the Dix‐Hallpike test and Epley maneuver, (b) accurately observe and document nystagmus patterns, and (c) always consider the possibility of other canals being involved when rendering a canal‐specific diagnosis and treatment for BPPV. Future clinical trials can investigate whether modifying the diagnostic approach to patients with dizziness may improve diagnostic accuracy and treatment outcomes.

## AUTHOR CONTRIBUTIONS

**Lisa Young:** Conceptualization; writing – original draft. **Shervin Badihian:** Conceptualization; data curation; formal analysis; writing – review and editing. **David S. Zee:** Conceptualization; data curation; formal analysis; writing – review and editing. **David E. Newman‐Toker:** Conceptualization; data curation; funding acquisition; writing – review and editing. **Kevin A. Kerber:** Conceptualization; data curation; writing – review and editing. **Anita Bhandari:** Conceptualization; data curation; formal analysis; software; writing – review and editing. **Rajneesh Bhandari:** Conceptualization; data curation; formal analysis; software; writing – review and editing.

## FUNDING INFORMATION

The patient reported in this manuscript was enrolled as part of the AVERT (Acute Video‐oculography for Vertigo in Emergency Rooms for Rapid Triage) clinical trial (clinicaltrial.gov identifier: NCT02483429). The AVERT clinical trial is funded by the National Institute on Deafness and Other Communication Disorders (NIDCD) (Grant number: U01DC013778), which provided support for Drs. Badihian, Newman‐Toker, and Zee in the initial assessment of this patient's clinical and video‐oculographic findings. No other grants, contracts, or other forms of financial support were used in the preparation of this manuscript.

## CONFLICT OF INTEREST STATEMENT

Dr. Newman‐Toker conducts research related to the diagnosis of dizziness and stroke, as well as diagnostic errors. He serves as the principal investigator for multiple grants and contracts on these topics, including the NIH‐sponsored AVERT clinical trial (NIDCD U01 DC013778, ClinicalTrials.gov #NCT02483429). Johns Hopkins has been loaned research equipment (video‐oculography [VOG] systems) by two companies for use in Dr. Newman‐Toker's research; one of these companies has also provided funding for research on diagnostic algorithm development related to dizziness, inner ear diseases, and stroke. Dr. Newman‐Toker has no other financial interest in these or any other companies. Dr. Newman‐Toker is an inventor on a provisional patent (US PCT/US2020/070304) for smartphone‐based stroke diagnosis in patients with dizziness. He gives frequent academic lectures on these topics and occasionally serves as a medico‐legal consultant for both plaintiff and defense in cases related to dizziness, stroke, and diagnostic error. Dr. Kevin Kerber receives publishing royalties from Oxford University Press. Dr. Anita Bhandari and Rajneesh Bhandari are directors of NeuroEquilibrium Diagnostic Systems Private Limited, India. All other authors have no conflicts of interest.

## ETHICS STATEMENT

The University of Michigan Institutional Review Board approved the study, which conforms to recognized standards established by the Declaration of Helsinki and the US Federal Policy for the Protection of Human Subjects.

## CONSENT

Written informed consent was obtained from the patient to publish this report in accordance with the journal's patient consent policy.

## Supporting information


Video S1.

Video S2.

Video S3.

Video S4.

## Data Availability

The authors confirm that the data supporting the findings of this study are available within the article and its supplementary materials.
